# Characterization of large volume subcutaneous injections using computed tomography imaging and simultaneous pressure measurements

**DOI:** 10.3389/fddev.2023.1223177

**Published:** 2023-07-13

**Authors:** Mona Purcell, Sahab Babaee, Michael Galluppi, John Cline, Guangli Hu, Ioan Petrescu, Jennifer Hughes, Meredith Allen, Eric Messina, Steven Persak, Yogita Krishnamachari, Ashley Lay-Fortenbery, Corin O. Miller

**Affiliations:** ^1^ Translational Imaging, Merck Research Laboratories, Merck and Co., Inc., Rahway, NJ, United States; ^2^ Device Development, Merck Research Laboratories, Merck and Co., Inc., Rahway, NJ, United States; ^3^ Laboratory Animal Resource, Merck Research Laboratories, Merck and Co., Inc., Rahway, NJ, United States; ^4^ Preclinical Development, Merck Research Laboratories, Merck and Co., Inc., Rahway, NJ, United States

**Keywords:** Subcutaneous, hyaluronidase, computed tomography, minipig, *in vivo*

## Abstract

Many commercially available biologics, previously delivered only intravenously, are being re-formulated for subcutaneous delivery to improve patient access and compliance. However, due to inherent solubility limitations, large volume injections (more than 2 mL) are typically required. Different strategies are being explored to improve the tolerability of such injections, including the co-formulation with hyaluronidase and/or implementing different needle designs. While there have been separate reports of measuring injection forces and using imaging to track injection delivery and tissue response, there is no current set of methods to simultaneously characterize the injection delivery (bleb) and measure injection pressures. In this study we describe the development of Computed Tomography imaging methods in minipigs to characterize the morphology of the bleb following injection, along with inline pressure measurements to assess subcutaneous pressure during injection using two different injection volumes, 4.5 mL and 9 mL. We show that these parameters change with injection volume, and that inclusion of hyaluronidase in the injection increases bleb dispersion and reduces skin distention while also lowering the injection pressure. This method will likely be a valuable tool for assessing and comparing different injection delivery methods and formulations.

## Introduction

Of the newly approved drugs from 2009–2017, 15.5% were biologics which is approximately twice the corresponding percentage of newly approved biologics from 2000–2008 (Batta, Kalra, & Khirasaria, 2020). Typically, the primary route of administration for biologics is intravenous (IV). While many of these therapies have had a marked impact on human health, the need for IV administration in a hospital setting limits patient access, convenience, and compliance. One attractive solution to this challenge is to modify the formulation to allow for subcutaneous (SC) delivery, as this type of administration can be performed in doctor’s offices, pharmacies, and potentially in the home by home-health aides or the patients themselves, greatly expanding patient access and adherence. Additionally, clinical studies have shown SC administration to be the preferred route of administration over IV, by both patients and heath care providers (Bittner, Richter, & Schmidt, 2018). The limited solubility of these large molecules, however, coupled with the need for sufficient plasma exposures lasting multiple months often requires the volume of such injections to be greater than 2 mL, which are generally referred to as “large volume SC injections”. For example, trastuzumab is formulated as a 5 mL SC injection while rituximab is formulated for 10–15 mL SC injection (Genentech, South San Francisco, CA). Such large volumes administered with standard needles generally cause patient discomfort, therefore different strategies are being implemented to improve upon this. One potential solution is to use a slow delivery of the injection, which can be accomplished either manually or with an auto-injector. The inclusion of hyaluronidase (HLN) in the injection formulation is an additional approach that has been employed (Frost, 2007; Buhren et al., 2016; Shi, Connor, Collins, & Kang, 2021). HLN is an enzyme that degrades hyaluronan (hyaluronic acid), a key component of the SC matrix and allows injection fluid to disperse more quickly within the SC space. For example, the commercial products Herceptin and Humira both have SC injection formulations which include HLN. Some of the more common adverse effects with the inclusion of HLN in formulations are injection site reaction, headache, fatigue, nausea, and fever. (Gilson & Zafar Gondal, 2023).

A key unmet need in the development of large volume SC injections is the ability to evaluate the performance of different injection devices and formulations in an animal model. For translational studies of SC or dermal injections, the pig is an ideal pre-clinical species and has been used extensively in recent years due to the similarity of its skin to humans (Qvist, Hoeck, Kreilgaard, Madsen, & Frokjaer, 2000; Mahl et al., 2006; Zheng et al., 2012; Stricker-Krongrad, Shoemake, & Bouchard, 2016). Properties such as the dermal to epidermal thickness ratio, epidermal turnover time, vascular anatomy, and the tight attachment of skin to subcutaneous connective tissue are similar in pigs and humans (Alex et al., 2020). In addition, minipigs such as the Gottingen used in this study, are especially favorable due to their slower growth curve and smaller size, making them easier to handle (Schuleri et al., 2008; Ganderup, Harvey, Mortensen, & Harrouk, 2012).

The application of imaging technologies to understand injections and the overall drug delivery process has increased recently. While preliminary studies were often done *ex-vivo* on excised tissue (Praestmark et al., 2012), more recent work has been done *in vivo* on larger species and in humans. For example, clinical MRI imaging was used to monitor the response to injection of Cabotegravir, an antiviral (Jucker et al., 2022), however the measurements derived from these images reflected the response of the affected tissue and not the injection process, *per se*. Computed Tomography (CT) imaging is well-suited to directly image the injection process. The minimal CT signal from the injection site (generally soft tissue) coupled with commercial CT contrast agents that can be formulated with HLN and/or therapeutic agents of interest produces images with dramatic contrast. In fact, CT imaging has been used in Yucatan minipigs to assess the effects of different injection rates and the inclusion of HLN on the morphology of the post injection fluid pocket (Connor, Taverna, Thrall, LaBarre, & Kang, 2020).

Finally, the characterization of the injection process would not be complete without a measurement of the pressure generated during and after the injection. While previous reports have measured injection forces and pressures under various conditions (Allmendinger et al., 2015; Verwulgen et al., 2018), these measurements have never been paired with images of the injection site itself to fully characterize the injection process. The goals of this study, therefore, were to develop simultaneous CT imaging and pressure measurement methods to characterize large-volume subcutaneous injections and to assess the effects of HLN on these injections.

## Methods

*Needles and Injections*–Since the use of metal needles during CT imaging would cause significant image artifacts, 3D printed, plastic resin needles (HTL, BMF Nano Material Technology Co., Maynard, MA.) with specifications identical to a standard 25G 1 ½” needle were manufactured by Boston Micro Fabrication (BMF) (Shenzhen City, Guangdong Province, China). HTL resin was chosen as its mechanical properties are superior to all the other resins in the BMF catalog, in addition to having greater accuracy and fine surface finish. All 3D-printed needles and extension tubing were sterilized with ethylene oxide prior to use.

The contrast agent Iohexol (Omnipaque 350 mg Iodine/mL, GE Healthcare, Inc) was used both alone and co-formulated at 750U/mL with 7.5% HLN which was shown to have little effect on the viscosity of the injection. Omnipaque was selected due to its high image contrast and similar viscosity (approx. 20 cP) to expected formulations of therapeutic agents. Viscosity measurements were performed using a RheoSense m-VROC II viscometer comparing different concentrations of the expected formulation with different concentrations of HLN at various shear rates.

*Animal Handling* - This study was reviewed and approved by Merck Institutional Animal Care and Use Committee, Merck & Co., Inc, Rahway, NJ, USA. Eight purpose-bred, naïve, litter-matched, female Gottingen miniature swine were obtained and allowed to acclimate for minimum of 7 days before receiving SC infusions. Animals were Panepinto sling-trained during acclimation period, ∼3 months old, and weighed 5–6 kg at the start of this 4-month study. Since the minipigs were scanned multiple times during the study, baseline, and post-CT blood samples were taken intermittently for CBC and clinical chemistry to monitor for thrombocytopenia, leukopenia, or other adverse effects. Animals were allowed to recover for a minimum of 5 days, but most often 10–14 days, before next imaging session.

Animals were overnight fasted prior to study procedures. On the day of study, minipigs were placed in a sling, administered Glycopyrrolate - (0.004–0.01 mg/kg, IM) to prevent hypersalivation and anesthetized with 4%–5% Isoflurane via nose cone. They were then intubated prior to being transported to the imaging laboratory. An I.V. catheter was placed for fluid maintenance. Isoflurane anesthesia was a maintained at 2%–3% based on heart rate. The animal was placed on a positive pressure medical grade ventilator at ∼12–14 respirations per minute and tidal pressure of 14–15 mmHg. A temperature probe, pulse oximeter, and end tidal CO2 monitor were connected. End tidal CO2 was maintained at 30 ± 5 mmHg. Body temperature was maintained by placing the animal on a K-module heating pad. The animal was positioned supine and head-first inside the camera gantry. The abdomen was shaved and cleansed with iodine prior to imaging.

Injection sites were pre-pierced subcutaneously using a standard 16G 1 ½” stainless steel needle. Multiple passes with this needle were required to create a sufficient insertion space for the 3D-printed needle. The 3D-printed needle was then inserted, and a drop of tissue glue was placed on injection site to decrease the chance of leakage. The injection was delivered using a Harvard Apparatus dual channel pump (10 mL/min) with either 4.5 or 9 mL of Omnipaque ± HLN. 100% and 10% Omnipaque fiducial markers were placed in the FOV. CT scans were acquired during the infusion at the following approximate time points: baseline, 5, 30, 60, 90, 120, 180, and 600 s. Breath frequency was decreased to 4 breaths/min with Pinsp value of 16 to simulate a ‘breath hold’ during the CT scans within first 3 min which was necessary for aligning pre- and post-injection images. Both standard needle and novel needle designs were evaluated with n = 3 repeats for Omnipaque ± HLN to properly assess injection parameters.

CT scanning was performed on a Biograph TruePoint 64 PET/CT camera (Siemens Healthcare, Erlangen, Germany). Scanning parameters of each scan were 160 mAs, 100 kV, 3.0 mm slice thickness, 0.5 s rotation, 1.25 pitch, and a reconstruction kernel of B60f sharp. Each scan delivered a radiation dose of 7.15 mGy which was well below limit for any haemopoietic changes in the animal, even accounting for multiple scans.

**FIGURE 1:**
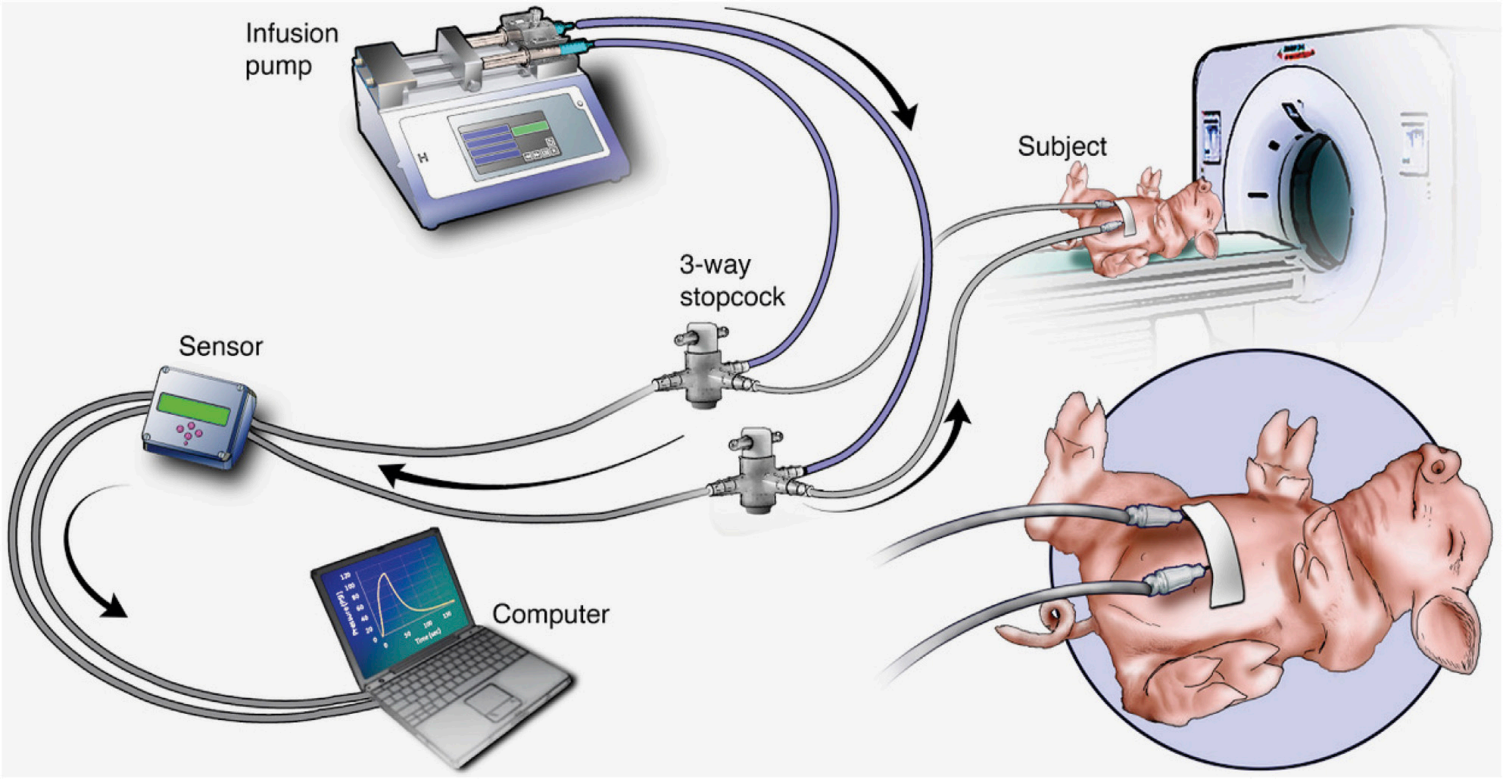
Experimental setup showing the minipig subject and all essential components.

The overall experimental setup is shown in Figure 1. This schematic the shows essential components needed for infusion, the placement of needles for SC delivery, and the pressure measurement equipment.

**FIGURE 2:**
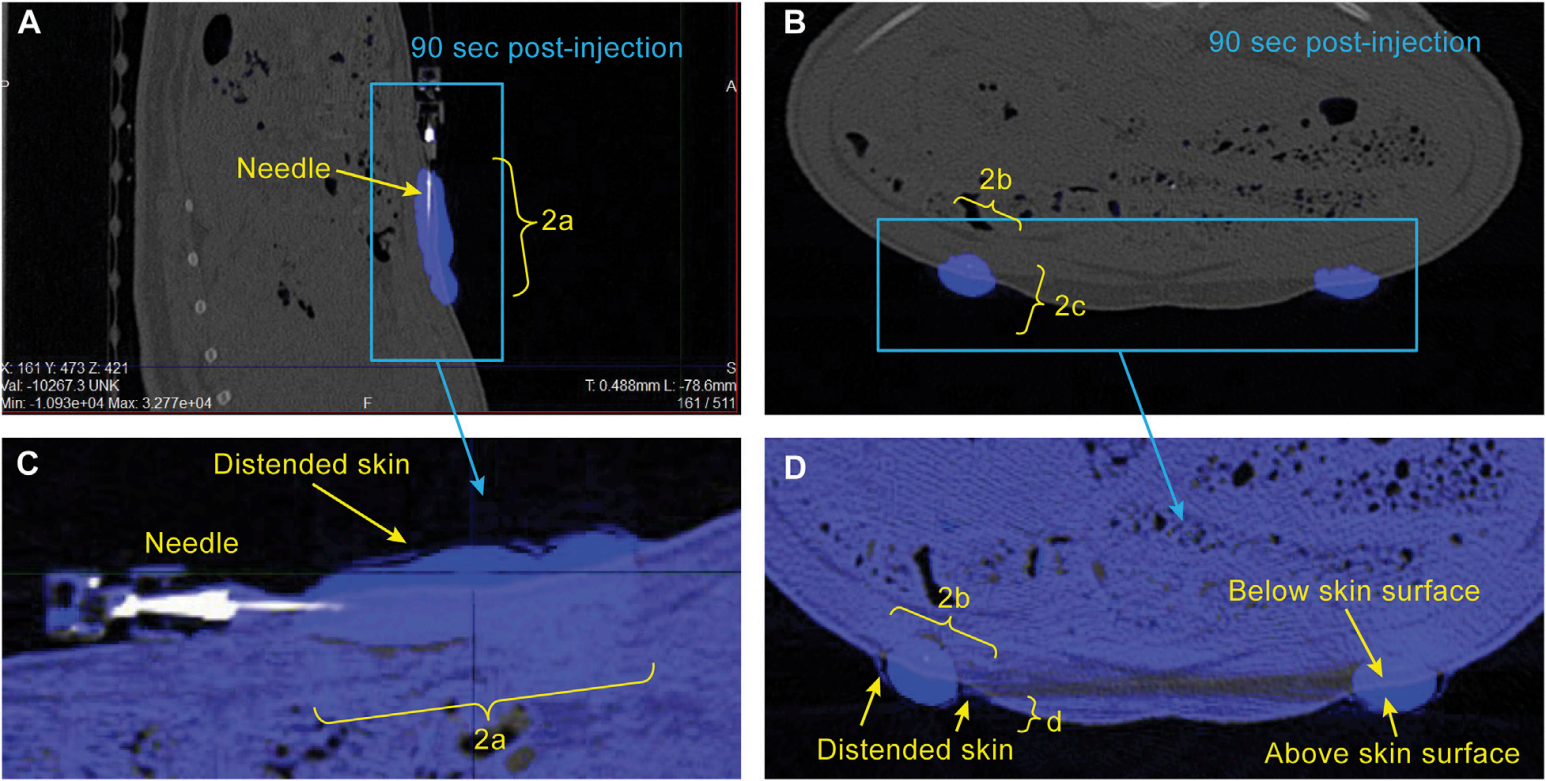
Example CT images showing sagittal **(A)** and axial **(B)** views of the bleb in the minipig abdomen 90 s following start of injection. Segmentation of the bleb (blue) is described in METHODS. Expanded views of the bleb-containing region of the image are shown in **(C, D)** with the post-injection image (blue) overlaid on the pre-injection image (grey) to illustrate the region where the skin has been distended above the original surface. The dimensions a, b, c, and d are used for estimation of bleb surface area and skin distention.

*CT Image analysis* - CT images were loaded into VivoQuant software (InVicro, Needham, MA) and the bleb was segmented using a nearest neighbor thresholding algorithm along with occasional manual touch-up (Figure 2). To assess injection dispersion the bleb was assumed to have an ellipsoidal shape, the dimensions of the bleb region were measured using the software, and the surface area of the bleb was calculated using the following formula:

S≈4πab1.6+ac1.6+bc1.6311.6

where a, b, and c are the half the distances across the bleb in each dimension (i.e., the length of the semi-axes shown in yellow brackets in Figures 2A, B).

To assess skin distension following the injection, pre- and post-injection images were overlaid and the dimensions of the portion of the bleb protruding above the original skin surface were measured manually (Figures 2C, D). The distended skin was assumed to have a half-ellipsoidal shape, and its volume was calculated using the following formula:

$$V\approx \frac{2}{3}\pi abc$$

*Pressure signal analysis* - SC pressure was monitored during and after injections using a pressure transducer (Honeywell 19C100 PG4K, DigiKey Electronics) interfaced with DAQami acquisition software and integrated into the injection lines via three-way stopcock. Pressure signals were sampled at a rate of 10 Hz and were imported into Matlab (MathWorks, Natick, MA) for offline analysis using in-house developed scripts. For each pressure recording the maximum pressure within 2 s of the cessation of the injection was detected, and the decay rate of the pressure during the 2 min following injection was measured by modelling with a double exponential fit and calculating the effective decay constant. These equations are shown below:

$$P\left(t\right)=A_{1}e^{-k_{1}t}+A_{2}e^{-k_{2}t}$$

$$k_{eff}=\frac{A_{1}k_{1}+A_{2}k_{2}}{A_{1}+A_{2}}$$

Where P(t) is the measured pressure vs. time, k_1_ and k_2_ are the two decay constants and A_1_ and A_2_ are the weights of each exponential portion of the fit.

## Results

Sample CT images acquired approximately 90 s post-injection start are shown in Figure 2 for a single minipig subject. The location of the bleb is straightforward due to the high image intensity conferred by the use of 100% Omnipaque. Also of note is the ability to detect the region of distended skin above baseline shown in Figures 2C, D. The excellent alignment of the grey (pre-injection) and blue (post-injection) overlaid images everywhere aside from the bleb region illustrates the importance of using a reduced respiration rate during image acquisition (described in METHODS), as this allows for visual detection of the distended region.

**FIGURE 3:**
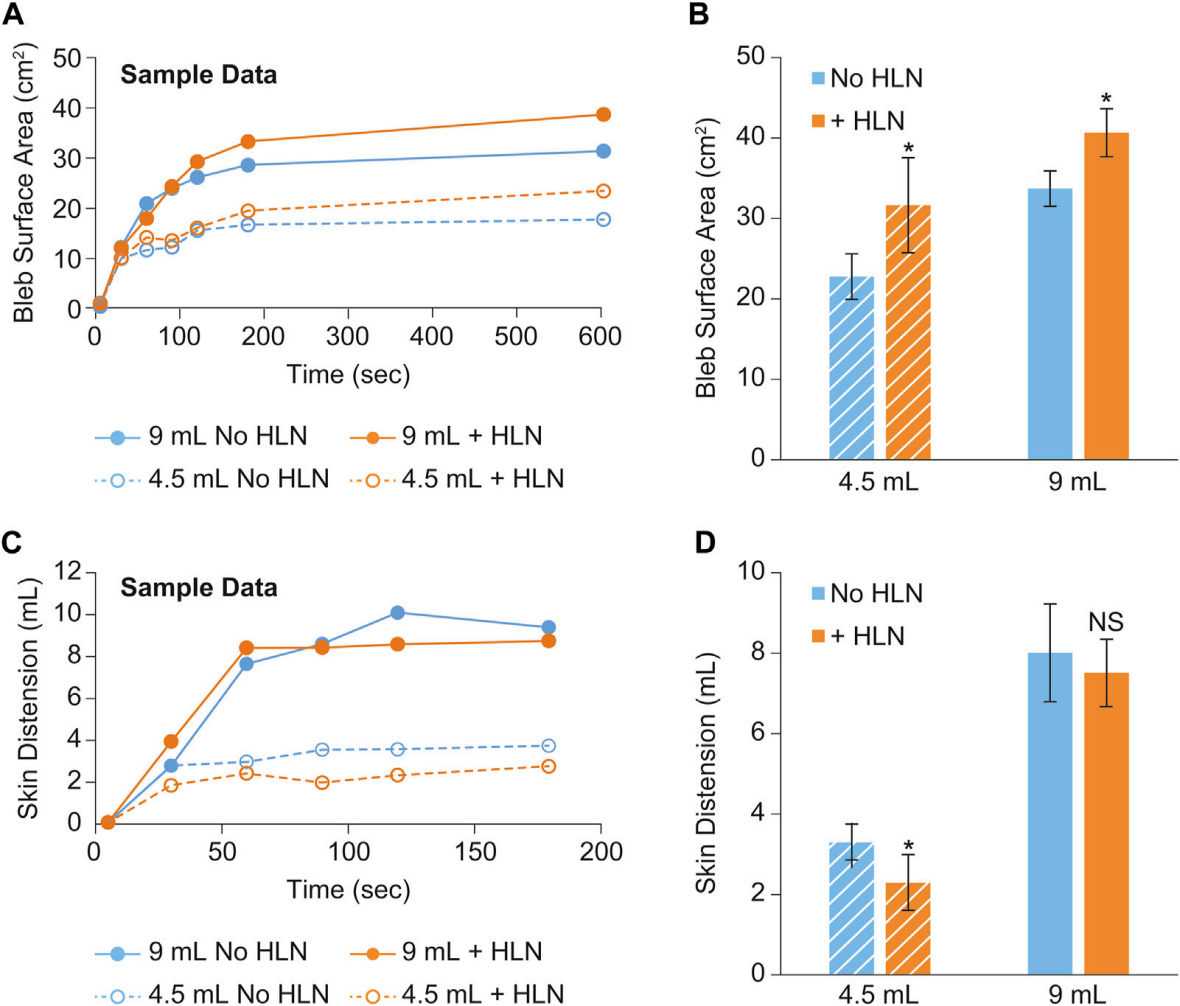
Example bleb surface area **(A)** and skin distention **(C)** data collected during a scanning session from a single minipig subject using two different injection volumes and with and without the inclusion of hyaluronidase in the injection. The bleb surface area 10 min post-injection and the skin distension 90–180 s post-injection are shown in **(B, D)**, respectively. The data were reported as mean ± s.d. of *n* = 3 measurements for each group. Two-sample Student’s t-tests were used to determine significance. *p < 0.05; NS, not significant.

Sample plots of bleb surface area and skin distention versus time are shown in Figures 3A, C, respectively. These data were collected from a single minipig subject using two different injection volumes ± HLN in the injection. Group means of the bleb surface area 10 min post-injection and the skin distension volume averaged over the 90–120 s post-injection are shown in Figures 3B, D, respectively. The inclusion of HLN produced either a significant increase or a trend for increase in bleb surface area (dispersion). In addition, the inclusion of HLN produced either a significant decrease or a trend for decrease in the skin distension during injection.

**FIGURE 4:**
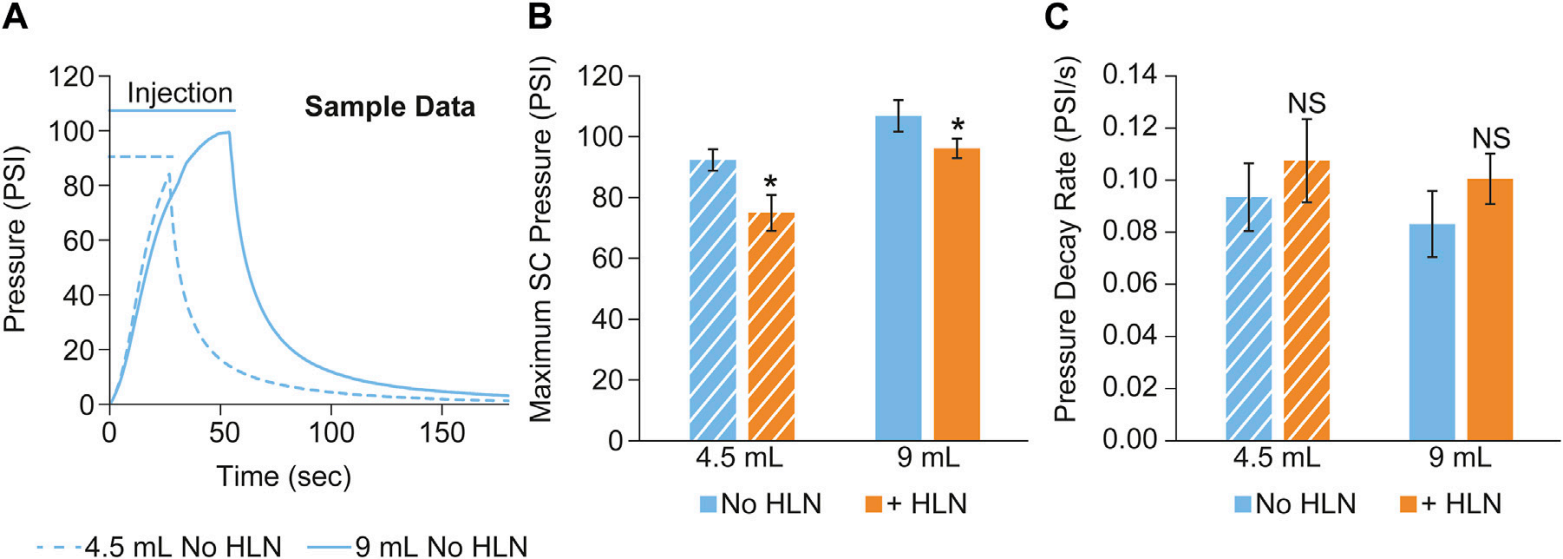
**(A)** Example subcutaneous pressure recordings collected during a scanning session from a single minipig subject using two different injection volumes. The maximum SC injection pressure and the pressure decay rate post-injection completion are shown in **(B, C)**, respectively. The data were reported as mean ± s.d. of *n* = 3 measurements for each group. Two-sample Student’s t-tests were used to determine significance. *p < 0.05; NS, not significant.

Sample subcutaneous pressure recordings collected during a scanning session from a minipig subject using two different injection volumes are shown in Figure 4A. Group means of the maximum injection pressure and the pressure decay rate (calculated as described in METHODS) post-injection completion are shown in Figures 4B, C, respectively. The inclusion of HLN produced a statistically significant decrease in the maximum injection pressure at both injection volumes, along with a trend for increased SC pressure relief rates following the injection.

## Discussion

*Experimental considerations–*The minipig is a widely accepted model for pre-clinical dermal studies, and while there are many similarities between the skin of the minipig and humans, there are important factors to consider for SC injections into the abdomen. One factor is the development of mammary tissue and vessels as minipigs age. Scientific data evaluating minipig mammary tissue development is limited (de Rijk et al., 2014), however it is not unreasonable to conclude that it increases when animals go into estrus (∼7–8 months). As the animals in our study aged, we observed an increase in irregular-shaped blebs. This may be due to an increase in mammary tissue and/or perhaps fibrous tissue development due to repeated injections.

Another challenge was the need to pre-pierce the injection site with a standard metal needle due to the fragility and inability of the 3D printed needles to puncture the skin. Several pass-throughs were required with the metal needle to enable the 3D printed needle to be inserted. This potentially created a hole slightly larger than if a direct insertion was able to be performed, and the need for tissue glue to prevent leakage.

*CT image analysis methods*–Two fundamental assumptions were made in the analysis of the CT images. Specifically, that the bleb shape was approximately ellipsoidal, and that the region of distended skin was approximately half-ellipsoidal. The first assumption allowed for bleb surface area to be *calculated* directly via measurement of the three cross-sectional axes rather than *approximated* via complicated 3D surface area algorithms. The majority of blebs observed had an overall regular shape with occasional jagged edges so we felt that former approach better reflected the overall dispersion of the bleb within the SC space and would be less subject to errors from interpolation between image slices and edge smoothing methods used in the latter approach. The region of skin distended during and following injection was best visualized via overlay of the pre- and post-injection images. Accordingly, it was difficult to develop thresholding algorithms to segment this region from a single image and so calculation of the distended skin volume using manual measurements of the three cross-sectional axes of the distended skin volume was the most convenient approach.

*Comparison with other publications*–While other reports have measured injection site morphology and injection force/pressure separately, this work presents the simultaneous measurement of important parameters related to both (dispersion, skin distension, and injection pressure). Our observed relationships of injection dispersion (surface area) versus time (Figure 3A) are qualitatively similar to those reported in Connor et al., but with reduced absolute values. This could also be due to differences in the viscosity of the injection as well as differences in the strain of minipig used, along with the small difference in injected volumes. Our injection pressure measurements were performed using an inline sensor split off from the injection line via 3-way stopcock which is different than those previously reported (Allmendinger et al., 2015; Verwulgen et al., 2018) where a force sensor attached to the syringe pump was used. While this resulted in a slower rise of pressure early in the part of the injection (cf Allmendinger et al., 2015; Figure 2), the maximum pressure values we observed were still consistent with those reported after converting to force (Newtons), and when comparing similar injection rates and viscosities.

*Relevance of injection parameters*–In this study we evaluated two different SC injection volumes of 4.5 mL and 9 mL, delivered over 27 and 54 s, respectively, with and without HLN. While there are no clinical reports on the relationship between SC pressure during injection and pain, we believe the conditions reported here likely reflect injections that would cause patient discomfort. For example, in previous studies, subjects reported increased pain with 3.5 mL SC injections compared to 1.2 mL injections (Dias, Abosaleem, Crispino, Gao, & Shaywitz, 2015). Similarly, SC injection of a 5 mL volume over approximately 5 min resulted in peak pain halfway through the injection (Woodley et al., 2021). Note that both these studies used injection volumes smaller than the maximum volume used in this study. The concentration of HLN included in the injections was also similar to commercial formulations and is expected to have minimal effect on overall pharmacokinetic parameters. For example, the effects of HLN on SC injection of human immunoglobulin were studied and PK parameters of the injections with and without HLN were found to be equivalent (Wasserman et al., 2012).

## Conclusion

This report focused on the development of imaging and pressure measurement methods to characterize SC injections using different injection volumes and the addition of HLN to produce varying injection conditions. These methods can be useful in the characterization of many delivery approaches such as novel needle designs (e.g., cross-drilled needles), formulations of varying viscosities, SC implants, dermal patches and needle-less syringes. Furthermore, these methods are translatable to human studies and could prove valuable in evaluating the relationship between injection pressure, bleb dispersion, skin distension and overall injection discomfort.

## Data Availability

The original contributions presented in the study are included in the article/Supplementary material, further inquiries can be directed to the corresponding author.
